# Black Ginseng Concentrate Restores Hair Loss-Associated Dysfunction in Human Follicle Dermal Papilla Cells

**DOI:** 10.3390/ijms27135889

**Published:** 2026-06-30

**Authors:** Jung Un Shin, Yun Hoo Jo, Minha Kim, Jungwon Min, Ki Soo Kim, Byeong Bae Jeon, Uk Sun Jung, Ki Hyun Kim, Eui Soon Kim, Chulwan Kim, Seung Hwan Lee, Dong Wook Shin

**Affiliations:** 1 Research Institute for Biomedical and Health Science, Konkuk University, Chungju 27478, Republic of Korea; ipd5252@kku.ac.kr (J.U.S.); julie3734@kku.ac.kr (Y.H.J.); melody2378@kku.ac.kr (M.K.); mjw0110@kku.ac.kr (J.M.); 2 Suwon R&D Center, Hanbit Flavor & Fragrance, 101-1511, Digital Empire 2, 88, Sinwonro, Yeongtong-gu, Suwon-si 16681, Republic of Korea; kskim@hffaroma.com (K.S.K.); bbjeon@hffaroma.com (B.B.J.); usjung@hffaroma.com (U.S.J.); reirai@hffaroma.com (K.H.K.); kimes7746@hffaroma.com (E.S.K.); 3 Department of Beauty Fragrance, Graduate School of Bio-Wellness Convergence, Konkuk University, Chungju 27478, Republic of Korea; chulwan98@kku.ac.kr (C.K.); leesh5856@kku.ac.kr (S.H.L.)

**Keywords:** hair loss, black ginseng concentrate, antioxidant, human follicle dermal papilla cells, mitochondrial function, anti-inflammation

## Abstract

Hair loss is closely associated with oxidative stress, which impairs the function of human follicle dermal papilla cells (HFDPCs) and disrupts hair follicle homeostasis. Current pharmacological treatments, such as minoxidil and finasteride, are effective but may cause adverse effects, highlighting the need for safer alternatives. In this study, we utilized a patented high-pressure processing method to produce black ginseng concentrate (BGC), which is significantly enriched with rare bioactive ginsenosides, including Rg3, Rg5, and Rk1, through optimized chemical transformation. We aimed to elucidate the protective effects of BGC against oxidative stress-induced damage in HFDPCs. BGC significantly reduced intracellular reactive oxygen species (ROS) levels. BGC also improved mitochondrial function, including an increased oxygen consumption rate (OCR). In addition, BGC activated hair growth-related signaling pathways by upregulating Wnt/β-catenin and increasing the phosphorylation levels of ERK and AKT. Collectively, these findings demonstrate that BGC protects HFDPCs from oxidative stress, improves mitochondrial function, and supports key signaling pathways associated with hair growth. This study suggests that BGC has potential as a natural agent for preventing oxidative stress-induced cellular dysfunction related to hair loss.

## 1. Introduction

Hair is a specialized skin appendage that plays important physiological and psychosocial roles, including protection against external stimuli, thermoregulation, and contribution to individual identity and social perception [1,2]. Each hair fiber is produced by a highly organized mini-organ known as the hair follicle, which resides within the dermis [3,4]. Located at the base of the follicle, the dermal papilla is a cluster of specialized mesenchymal cells that serve as a critical regulatory center for hair formation [5]. Dermal papilla cells interact dynamically with surrounding epithelial matrix cells to coordinate hair shaft production and follicular growth [6,7]. Crucially, the maintenance of the anagen phase entails high metabolic demand; recent evidence suggests that mitochondrial dysfunction serves as a primary trigger for the premature anagen-to-catagen transition. This highlights the vital importance of bioenergetic integrity in DPCs for sustaining hair growth [8,9]. Hair follicles undergo a tightly regulated cyclic process consisting of the growth phase (anagen), regression phase (catagen), and resting phase (telogen) [10,11]. The maintenance of normal hair cycling depends largely on the functional integrity of dermal papilla cells, which provide essential signaling cues to sustain follicular activity [12,13,14]. Emerging evidence suggests that the disruption of cellular homeostasis within the dermal papilla, particularly under conditions of oxidative stress, can impair these regulatory interactions and compromise the capacity of the follicle to maintain active growth [15]. Thus, the preservation of dermal papilla cell function is fundamental to sustaining active hair growth and mitigating processes that contribute to hair loss.

Hair loss results from a multifactorial interplay of genetic predisposition, hormonal imbalance, aging, environmental exposure, and inflammatory processes [16,17]. Increasing evidence highlights oxidative stress as a central contributor to hair follicle dysfunction [18,19]. Reactive oxygen species (ROS) are naturally generated as byproducts of cellular metabolism [20,21,22]. However, under pathological conditions, their excessive accumulation disrupts redox balance [23,24]. Increased oxidative stress has been observed in the scalp of individuals with hair loss [25,26]. Excess ROS can impair the viability and function of dermal papilla cells [27]. Beyond inducing direct molecular damage, oxidative stress can disrupt mitochondrial homeostasis [28,29]. This disruption reduces cellular energy production and impairs metabolic activity, processes that are essential for sustaining active hair growth [30,31]. Such alterations may interfere with intracellular signaling pathways required for maintenance of the anagen phase and promote follicular regression [32,33]. To better understand how oxidative stress contributes to hair growth impairment at the cellular level, experimental systems that recapitulate dermal papilla dysfunction have been widely employed [34]. Because dermal papilla cells serve as key regulators of hair follicle activity, oxidative stress-induced dysfunction of these cells is commonly utilized as an in vitro model for investigating the molecular mechanisms underlying hair growth impairment [35,36,37]. Hydrogen peroxide (H_2_O_2_), a representative ROS, is commonly employed to induce controlled oxidative stress in dermal papilla cells, thereby mimicking stress-associated follicular dysfunction [38,39].

Current pharmacological treatments for hair loss, including minoxidil and 5α-reductase inhibitors, primarily target vascular modulation or androgen metabolism [40,41]. While these agents can slow hair loss progression or stimulate regrowth in certain individuals, their therapeutic effects are often partial and require continuous use to maintain efficacy [42]. Furthermore, they largely overlook the fundamental restoration of redox homeostasis and bioenergetic integrity within the DPC microenvironment [43]. Given the emerging recognition of oxidative damage as a contributing factor in hair follicle impairment, there is a growing need for alternative or complementary strategies that more directly target redox imbalance and cellular functional decline [44,45].

*Panax ginseng* C.A. Meyer (*P. ginseng*) has long been used in traditional medicine owing to its diverse biological activities [46,47]. Among its processed forms, black ginseng is produced through a traditional method known as “Gujeung Guopo,” which involves nine cycles of steaming and drying [48,49,50]. This repetitive processing induces the transformation of common ginsenosides into rare metabolites (e.g., Rg3, Rg5, and Rk1), which exhibit superior membrane permeability and more potent bioactivity compared to those found in white or red ginseng, offering a distinct advantage for topical and cellular target modulation [51,52]. Several of these transformed compounds have been reported to exhibit antioxidant and cytoprotective properties under oxidative stress conditions [53,54,55]. While black ginseng is traditionally prepared through these nine cycles, we have overcome the limitations of this time-consuming process by developing a proprietary and patented production platform (Korean Patent No. 10-2295116). This innovation uniquely integrates High-Pressure Processing (HPP) to maximize the efficiency of ginsenoside conversion within significantly fewer cycles while ensuring a standardized high concentration of bioactive constituents.

The present study specifically tests the hypothesis that this uniquely processed BGC acts as a multi-target agent that not only neutralizes ROS but also actively recalibrates the Wnt/β-catenin signaling axis by restoring mitochondrial respiratory capacity in human follicle dermal papilla cells (HFDPCs). Using H_2_O_2_-induced oxidative injury in HFDPCs as a model [56], we evaluated the capacity of BGC to attenuate intracellular ROS accumulation, restore mitochondrial bioenergetics, and consequently modulate hair growth-associated molecular signaling under oxidative stress conditions. Biotin, a nutrient widely recognized for its role in maintaining hair health, was included as a positive control to provide a comparative reference for the observed cellular responses [57,58,59]. Through comprehensive cellular and molecular analyses, this study aims to clarify the role of BGC in attenuating oxidative stress-induced dermal papilla cell dysfunction and to provide mechanistic insights into hair growth-associated cellular processes.

## 2. Results

### 2.1. Cell Viability of BGC in HFDPCs

**Figure 1:**
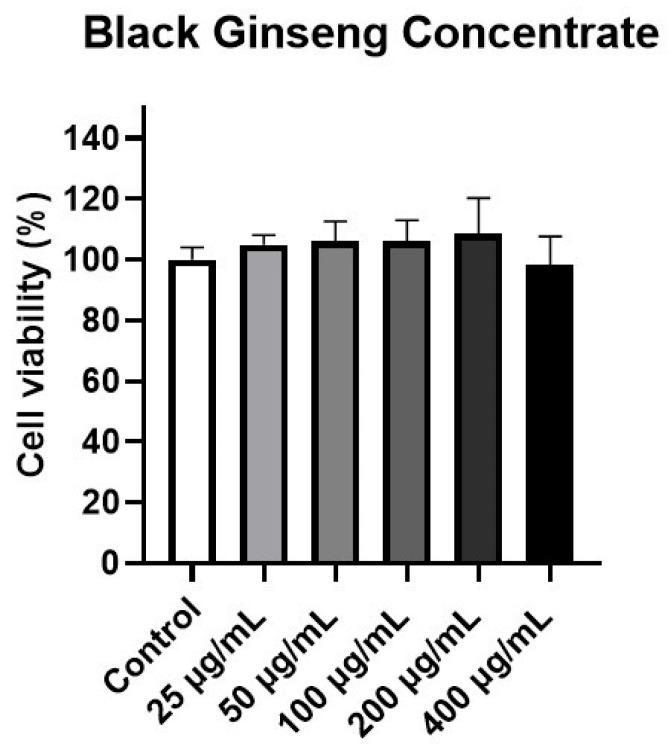
Cell viability of BGC in HFDPCs. Cell viability at different BGC concentrations was evaluated using the EZ-cytox assay and expressed as a percentage relative to the untreated control group. Data are presented as the mean ± SD (*n* = 3). No significant difference was observed between the control group and the BGC-treated groups.

To evaluate the potential cytotoxic effects of BGC on HFDPCs, cell viability was determined using the EZ-cytox assay. Cells were treated with increasing concentrations of BGC (25, 50, 100, 200, and 400 μg/mL) for 24 h. Cell viability remained above 100% even at 200 ppm BGC (Figure 1), indicating no significant cytotoxicity at this concentration. A preliminary dose–response analysis was performed, and 25 and 50 μg/mL were selected as the primary concentrations for subsequent experiments based on their consistent biological effects. Therefore, 50 μg/mL BGC was selected as the maximum concentration for subsequent experiments.

### 2.2. BGC Enhanced Cell Migration in H_2_O_2_-Induced Damaged HFDPCs

**Figure 2:**
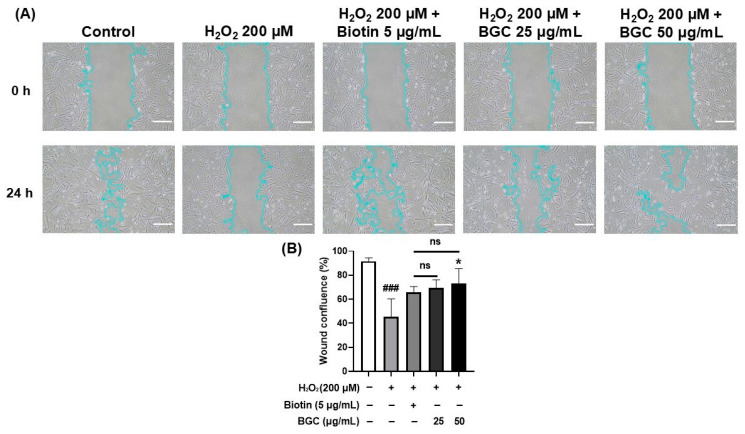
BGC enhances the migratory capacity of H_2_O_2_-treated HFDPCs. Cells were co-treated with 200 μM H_2_O_2_ and either 5 μg/mL biotin or BGC (25 and 50 μg/mL) for 24 h. (**A**) Representative images were obtained using a phase-contrast microscope (scale bar, 50 μm). Images are representative of three independent experiments. (**B**) Quantitative analysis of the wound-closure area was conducted using ImageJ software (version 1.53e). Values represent the mean ± SD from three independent biological experiments (*n* = 3). Statistical significance is indicated as follows: ns, not significant; * *p* < 0.05 compared with the H_2_O_2_-treated group; and ^###^ *p* < 0.001 compared with the control group. No significant difference was observed between the biotin-treated group and the BGC-treated group.

The migratory capacity of HFDPCs is essential for maintaining hair growth and regenerative activity [60]. Oxidative stress induced by H_2_O_2_ disrupts dermal papilla cell homeostasis and impairs migratory function, which is closely associated with hair growth impairment [61]. Therefore, a wound healing assay was performed to investigate whether BGC could restore cell migration in H_2_O_2_-induced damaged HFDPCs. In our previous study, we optimized the H_2_O_2_ concentration at 200 μM for a 24 h treatment period to effectively induce cellular dysfunction without excessive cell death [62]. Biotin was employed as a positive control at a concentration of 5 μg/mL, which was determined to be both non-cytotoxic and biologically effective based on preliminary cell viability and functional assays (Figure S1). After 24 h, the H_2_O_2_-treated group exhibited significantly reduced cell migration compared with the control group. In contrast, BGC treatment markedly enhanced wound closure at concentrations of 25 and 50 ug/mL (Figure 2A,B), whereas no restorative effects were observed at higher doses of 100 and 200 ug/mL (Figure S2). Compared with the H_2_O_2_-treated group, BGC treatment increased wound closure by 163.3 ± 50.9% and 183.9 ± 105.8% at 25 and 50 μg/mL, respectively, indicating a consistent trend toward enhanced wound closure was evident following BGC treatment. In addition, BGC at concentrations of 50 μg/mL or lower alone did not significantly alter cell migration under basal conditions (Figure S3).

### 2.3. BGC Restored ALP Activity in HFDPCs Exposed to H_2_O_2_

**Figure 3:**
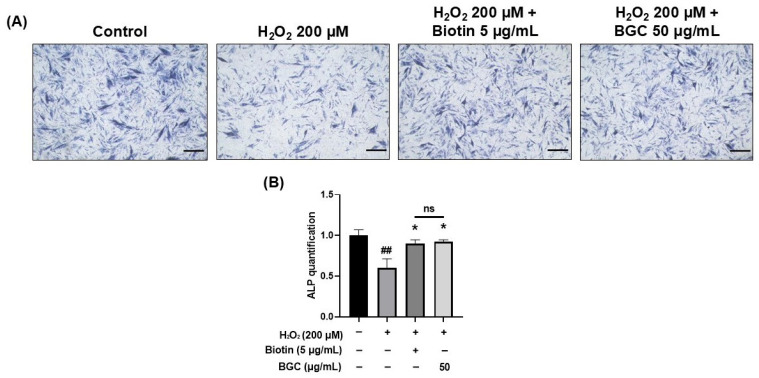
Effects of BGC on ALP expression in H_2_O_2_-induced HFDPCs. An ALP assay was conducted in HFDPCs exposed to 200 µM H_2_O_2_. Cells were pre-treated with 5 μg/mL biotin or 50 μg/mL BGC in the presence of 200 μM H_2_O_2_ for 24 h. (**A**) Representative phase-contrast images showing ALP expression in HFDPCs (scale bar, 50 µm). The images displayed represent three independent experiments. (**B**) ALP expression levels were quantified using ImageJ software (version 1.53e) to provide objective data. Values represent the mean ± SD from three independent biological experiments (*n* = 3). Statistical significance is indicated as follows: ns, not significant; * *p* < 0.05 compared with the H_2_O_2_-treated group; and ^#*#*^ *p* < 0.01 compared with the control group. No significant difference was observed between the biotin-treated group and the BGC-treated group.

Alkaline phosphatase (ALP), a representative marker of HFDPCs, is widely recognized as a key indicator of hair growth-promoting activity [63,64]. Previous studies have demonstrated that the hair-inductive capacity of HFDPCs is closely associated with ALP activity [65,66]. As expected, treatment with 5 μg/mL biotin, used as a positive control, significantly increased ALP expression compared with the H_2_O_2_-treated group. Treatment with 50 μg/mL BGC also increased ALP expression compared with the H_2_O_2_-treated group (Figure 3A,B). Compared with the H_2_O_2_-treated group, BGC treatment restored ALP activity by 158.4 ± 36.1%.

### 2.4. BGC Attenuated H_2_O_2_-Induced Oxidative Stress in HFDPCs

**Figure 4:**
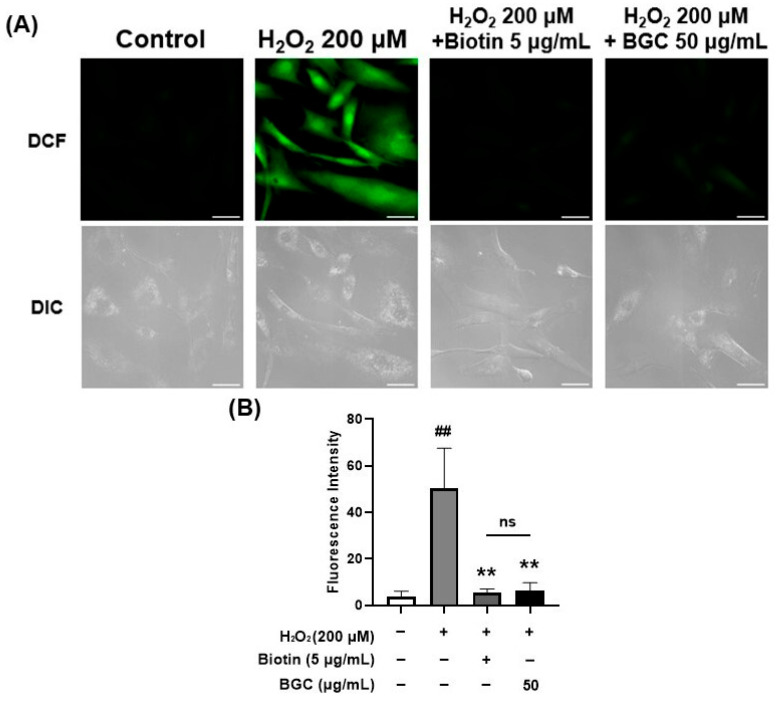
BGC mitigated intracellular ROS in H_2_O_2_-Damaged HFDPCs. Cells were pretreated with 5 μg/mL biotin or 50 μg/mL BGC for 22 h and then co-treated with 200 μM H_2_O_2_ for 2 h. (**A**) Representative DCF-DA fluorescence images showing intracellular ROS accumulation (DCF, green fluorescence, FITC channel) along with DIC images for cell morphological reference (scale bar, 50 μm). (**B**) Quantitative analysis of relative fluorescence intensity was performed using ImageJ software (version 1.53e). Values represent the mean ± SD from three independent biological experiments (*n* = 3). Statistical significance is indicated as follows: ns, not significant; ** *p* < 0.01 compared with the H_2_O_2_-treated group; and ^##^ *p* < 0.01 compared with the control group. No significant difference was observed between the biotin-treated group and the BGC-treated group.

Intracellular accumulation of ROS in HFDPCs is known to impair cellular homeostasis and contribute to hair growth-related dysfunction [67]. To evaluate the antioxidant effects of BGC, intracellular ROS levels were measured using the DCF-DA assay. H_2_O_2_ treatment markedly increased ROS production compared with the control group, whereas treatment with 50 μg/mL BGC significantly reduced ROS accumulation (Figure 4A,B). In contrast, treatment with 50 μg/mL BGC in the absence of H_2_O_2_ did not significantly affect intracellular ROS levels (Figure S4). Compared with the H_2_O_2_-treated group, BGC treatment reduced intracellular ROS by 12.4 ± 6.0%. These findings suggest that BGC reduces intracellular ROS under oxidative stress conditions.

### 2.5. BGC Improved Mitochondrial Membrane Potential in H_2_O_2_-Damaged HFDPCs

**Figure 5:**
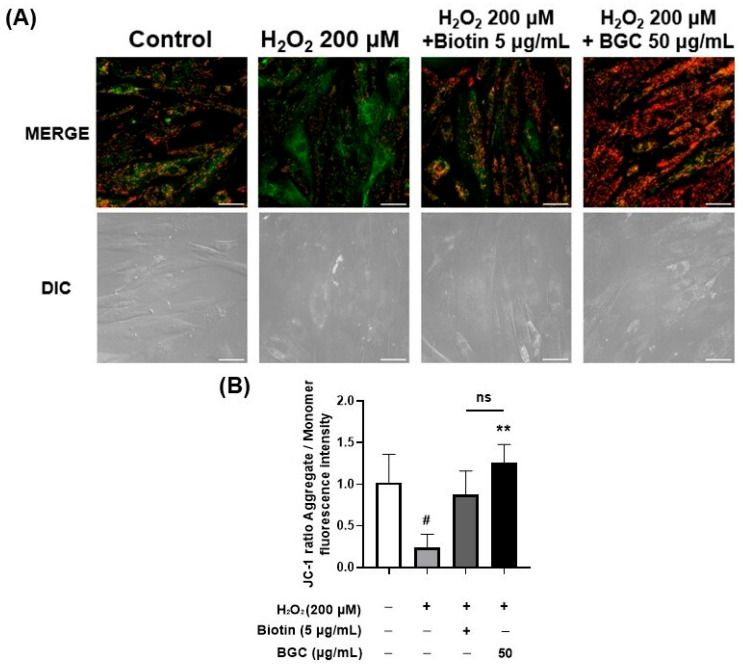
BGC improved mitochondrial membrane potential in H_2_O_2_-damaged HFDPCs. Cells were pretreated with 5 μg/mL biotin or 50 μg/mL BGC for 22 h, and then exposed to 200 μM H_2_O_2_ for 2 h. (**A**) Representative JC-1 images showing green fluorescence (depolarized mitochondria) and red fluorescence (polarized mitochondria) (scale bar, 50 μm). The images are representative of three independent trials. (**B**) The relative fluorescence intensity was determined via ImageJ software (version 1.53e). Values represent the mean ± SD from three independent biological experiments (*n* = 3). Statistical significance is indicated as follows: ns, not significant; ** *p* < 0.01 compared with the H_2_O_2_-treated group; and ^#^ *p* < 0.05 compared with the control group. No significant difference was observed between the biotin-treated group and the BGC-treated group.

Mitochondrial function is essential for the regulation of dermal papilla cell activity and hair follicle cycling [28,68]. To evaluate mitochondrial membrane potential, JC-1 staining was performed in HFDPCs. JC-1 emits red fluorescence in polarized mitochondria and green fluorescence in depolarized mitochondria. As shown in the JC-1 images, H_2_O_2_ treatment markedly increased green fluorescence, indicating mitochondrial membrane depolarization. Conversely, treatment with BGC restored mitochondrial membrane potential, as evidenced by the predominance of red fluorescence compared with the H_2_O_2_-treated group (Figure 5A,B). Compared with the H_2_O_2_-treated group, BGC treatment improved mitochondrial membrane potential by 420.6 ± 55.8%.

### 2.6. BGC Enhanced Adenosine Triphosphate (ATP) Production in H_2_O_2_-Damaged HFDPCs

**Figure 6:**
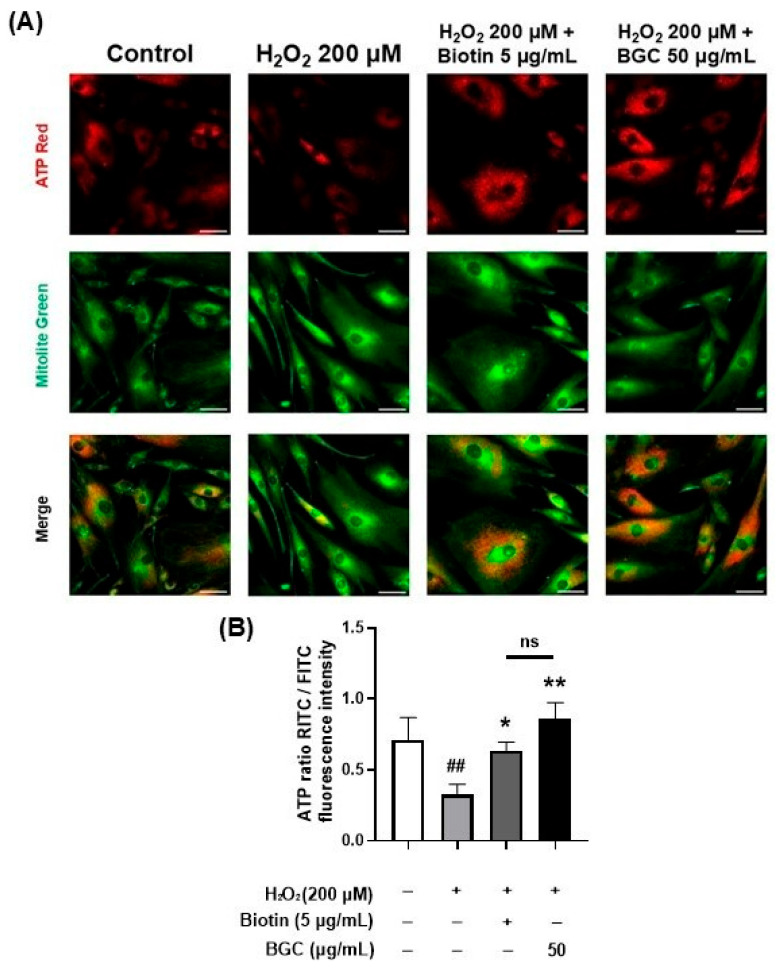
BGC promoted ATP production in H_2_O_2_-Damaged HFDPCs. Cells were pretreated with 5 μg/mL biotin or 50 μg/mL BGC for 22 h, and then exposed to 200 μM H_2_O_2_ for 2 h. (**A**) Representative images from the live-cell ATP assay. Red fluorescence indicates ATP levels, and green fluorescence indicates mitochondria (scale bar, 50 μm). The images are representative of three independent trials. (**B**) Quantitative analysis of ATP-to-mitochondria fluorescence intensity was performed using ImageJ software (version 1.53e). Values represent the mean ± SD from three independent biological experiments (*n* = 3). Statistical significance is indicated as follows: ns, not significant; * *p* < 0.05, ** *p* < 0.01 compared with the H_2_O_2_-treated group; and ^##^ *p* < 0.01 compared with the control group. No significant difference was observed between the biotin-treated group and the BGC-treated group.

Mitochondrial ATP production is essential for maintaining dermal papilla cell activity, which supports hair growth and cycling [8]. Based on the JC-1 results indicating that BGC restored mitochondrial membrane potential, we further evaluated mitochondrial ATP production in HFDPCs using a live-cell ATP assay. H_2_O_2_ treatment markedly reduced ATP production relative to the control group, indicating mitochondrial dysfunction under oxidative stress conditions. By contrast, BGC treatment considerably increased ATP production relative to the H_2_O_2_-treated group (Figure 6A,B). Compared with the H_2_O_2_-treated group, BGC treatment enhanced ATP production by 278.9 ± 97.0%. These results suggest that BGC restores mitochondrial function in HFDPCs.

### 2.7. BGC Increased Cellular Oxygen Consumption in H_2_O_2_-Damaged HFDPCs

**Figure 7:**
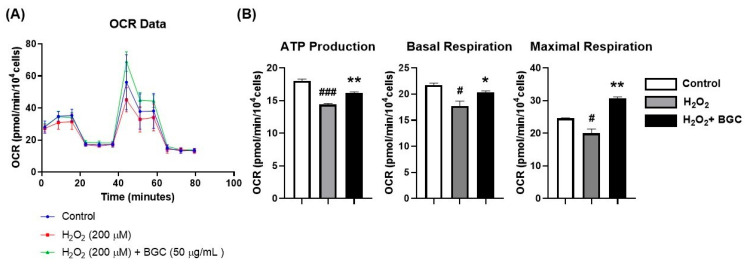
BGC Restored Oxygen Consumption Rate in H_2_O_2_-Damaged HFDPCs. The oxygen consumption rate of HFDPCs was measured using a Seahorse XF analyzer. Cells were co-treated with 50 μg/mL BGC and 200 μM H_2_O_2_ for 45 min. (**A**) Oxygen consumption rate profiles of HFDPCs. (**B**) Quantification of ATP production, basal respiration, and maximal respiration was calculated from the OCR profiles. Values represent the mean ± SD from three independent biological experiments (*n* = 3). Statistical significance was indicated as * *p* < 0.05, ** *p* < 0.01 compared with the H_2_O_2_-treated group and ^#^ *p* < 0.05, ^###^ *p* < 0.001 compared with the control group.

Cellular energy metabolism is closely linked to mitochondrial respiratory activity in HFDPCs [69]. The oxygen consumption rate (OCR) is commonly used to evaluate mitochondrial respiration and overall metabolic activity [70]. Based on the mitochondrial functional changes observed in the JC-1 and ATP assays. We further examined mitochondrial respiratory capacity by measuring OCR. H_2_O_2_ treatment significantly reduced multiple mitochondrial respiratory parameters, including basal respiration, maximal respiration, and ATP production, compared with the control group. In contrast, treatment with BGC alleviates oxidative stress-induced mitochondrial dysfunction and restores mitochondrial respiratory activity in HFDPCs (Figure 7A,B). Compared with the H_2_O_2_-treated group, BGC treatment enhanced APT production by 112.4 ± 0.4%, basal respiration by 115.6 ± 7.7%, and maximal respiration by 153.8 ± 12.6%.

### 2.8. BGC Activated Wnt/β-Catenin and ERK/AKT Signaling in H_2_O_2_-Damaged HFDPCs

**Figure 8:**
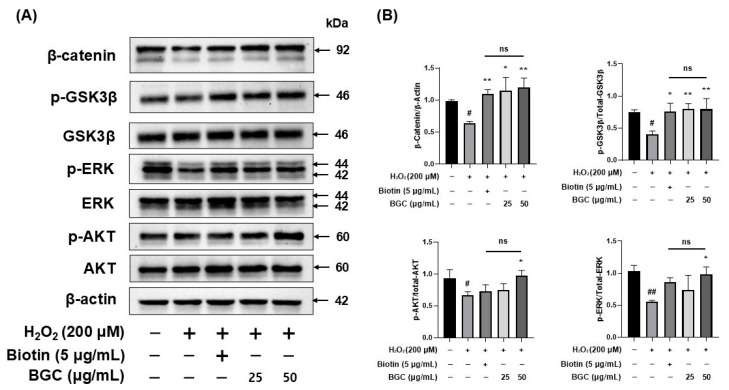
BGC Increased β-Catenin Level and Phosphorylation Levels of ERK, GSK3β, and AKT in H_2_O_2_-Damaged HFDPCs. Cells were pre-treated with 5 μg/mL biotin or 50 μg/mL BGC for 22 h and then treated with 200 μM H_2_O_2_ for 2 h. (**A**) Representative Western blot images showing the relative expression levels of each protein. (**B**) Relative expression levels of each protein are shown. Values represent the mean ± SD from three independent biological experiments (*n* = 3). Statistical significance is indicated as follows: ns, not significant; * *p* < 0.05, ** *p* < 0.01 relative to the H_2_O_2_-treated group. *^#^ p* < 0.05, and *^##^ p* < 0.01 compared with the control group. No significant difference was observed between the biotin-treated group and the BGC-treated group.

Wnt/β-Catenin signaling, together with ERK and AKT pathways, plays an important role in regulating the biological activity and hair growth-related functions of HFDPCs [71]. Oxidative stress has been reported to disrupt these signaling networks, leading to impaired cellular function [72]. To determine whether BGC modulates these pathways under oxidative stress, the expression level of β-Catenin and the phosphorylation levels of ERK, GSK3β, and AKT were analyzed by Western blotting. H_2_O_2_ treatment markedly reduced β-Catenin expression and decreased the phosphorylation levels of ERK, GSK3β, and AKT compared with the control group. However, BGC treatment significantly upregulated β-Catenin expression and restored the phosphorylation levels of ERK, GSK3β, and AKT in H_2_O_2_-treated HFDPCs (Figure 8A,B). These results indicate that BGC counteracts oxidative stress-induced suppression of key signaling pathways involved in HFDPC function.

### 2.9. BGC Recovered β-Catenin Expression in H_2_O_2_-Damaged HFDPCs in a 3D Spheroid Model

**Figure 9:**
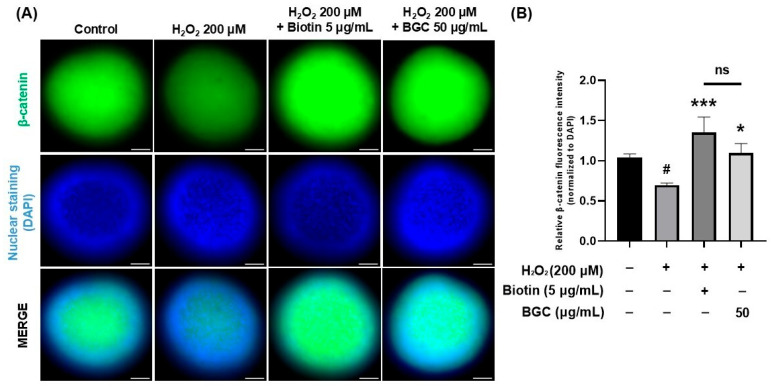
BGC promoted β-Catenin expression in H_2_O_2_-Damaged HFDPC spheroid. HFDPCs were cultured as 3D spheroids to mimic the follicle-like microenvironment. Spheroids were pretreated with 5 μg/mL biotin or 50 μg/mL BGC for 22 h, and then treated with 200 μM H_2_O_2_ for 2 h. (**A**) Representative images of immunofluorescence staining of β-Catenin in HFDPC spheroids (scale bar, 50 μm). The images are representative of three separate trials. (**B**) Quantification of β-Catenin fluorescence intensity normalized to DAPI staining (β-Catenin/DAPI). Values represent the mean ± SD from three independent biological experiments (*n* = 3). Statistical significance is indicated as follows: ns, not significant; * *p* < 0.05, *** *p* < 0.001 compared with the H_2_O_2_-treated group; and ^#^ *p* < 0.05 compared with the control group. No significant difference was observed between the biotin-treated group and the BGC-treated group.

HFDPCs were cultured as three-dimensional (3D) spheroids to better recapitulate the follicular microenvironment compared with conventional two-dimensional (2D) monolayer cultures [73,74]. The 3D spheroid model preserves cell–cell interactions and spatial architecture, allowing a more physiologically relevant evaluation of hair-related signaling [75]. Spheroid diameter was measured to assess morphological changes after treatment. No significant differences in spheroid size were observed among the groups (Figure S5). β-Catenin expression was then examined by immunofluorescence staining. H_2_O_2_ exposure reduced β-Catenin levels compared with the control. BGC treatment increased β-Catenin expression relative to the H_2_O_2_-treated group. These findings suggest that BGC alleviates oxidative stress-induced suppression of Wnt/β-Catenin signaling in HFDPC spheroids (Figure 9A,B). Compared with the H_2_O_2_-treated group, BGC treatment recovered β-Catenin expression 157.7 ± 22.0%.

## 3. Discussion

The proper function of HFDPCs is critical for maintaining cellular homeostasis, proliferation, and hair-inductive potential [76,77]. Oxidative stress, such as that induced by H_2_O_2_, impairs HFDPC viability, migration, and functional markers, contributing to hair loss-related dysfunction [62].

Our data indicate that BGC enhances HFDPC antioxidant defenses, proliferation, and functional recovery. H_2_O_2_ treatment impaired cell migration and increased intracellular ROS levels, while BGC significantly reduced ROS accumulation (Figure 4A,B) and promoted migration (Figure 2A,B), suggesting a direct link between the antioxidant properties of BGC and the restoration of cellular motility. Consistently, ALP activity, a key marker of HFDPC functionality, was restored to levels comparable to the control group following BGC treatment (Figure 3A,B).

Mitochondrial function and cellular energy metabolism are essential for sustaining HFDPC activity [78]. JC-1 staining showed that BGC treatment attenuated H_2_O_2_-induced mitochondrial depolarization (Figure 5A,B). In parallel, intracellular ATP levels were significantly increased in the BGC-treated group, suggesting improved cellular energy metabolism (Figure 6A,B). OCR analysis further confirmed that BGC enhanced mitochondrial respiration relative to H_2_O_2_-treated cells (Figure 7A,B). The restoration of ATP levels (Figure 6) and OCR (Figure 7) was not an independent event but was strictly coupled with the stabilization of the mitochondrial membrane potential (Figure 5), which was initially protected by the ROS-scavenging activity of BGC (Figure 4). These results demonstrate that BGC preserves mitochondrial integrity and metabolic activity under oxidative stress, ensuring sufficient energy supply to support proliferation, migration, and functional maintenance. Previous studies on black ginseng-derived components have largely focused on their general antioxidant properties in skin cells [79,80,81]. In contrast, this study demonstrates for the first time that BGC directly rescues the metabolic flexibility of HFDPCs by restoring mitochondrial respiration and ATP production under oxidative stress. This metabolic restoration acts as a prerequisite for the stabilization of β-catenin, providing a more comprehensive understanding of its hair-loss-inhibiting potential.

Beyond general cellular metabolism, hair growth-related signaling pathways are crucial for HFDPC functionality [82]. Our data indicate that the alleviation of oxidative stress (Figure 4) and the restoration of mitochondrial function (Figure 5, Figure 6 and Figure 7) act as a prerequisite for the activation of ERK/AKT and Wnt/β-catenin signaling. In HFDPCs, excessive ROS is known to trigger the “off-state” of these survival pathways. By scavenging ROS and stabilizing the mitochondrial membrane potential, BGC creates a cellular environment conducive to signaling transduction. Furthermore, the activation of ERK/AKT is well-documented to promote downstream anti-apoptotic and antioxidant protein expression, creating a positive feedback loop that further enhances mitochondrial repair [77,83]. These pathways are not independent phenomena but are integrated into a coordinated cellular response initiated by BGC’s primary antioxidant action.

Western blot analysis revealed dose-dependent upregulation of β-catenin, GSK3β, ERK, and AKT in BGC-treated cells compared with only H_2_O_2_-treated cells (Figure 8). It is well-established that the phosphorylation of GSK3β at Ser9 leads to its inactivation, which in turn prevents the degradation of β-catenin, leading to its stabilization and nuclear translocation. Therefore, our data showing the increase in GSK3-β (Ser9) and the subsequent rise in β-catenin levels (Figure 8) provide direct and sufficient evidence for the regulatory mechanism of the Wnt/beta-catenin signaling pathway by BGC.

In 3D spheroid cultures, immunofluorescence confirmed enhanced β-catenin expression, demonstrating that BGC supports both molecular signaling and the structural organization of HFDPC aggregates (Figure 9A,B). These observations suggest that BGC promotes hair-inductive signaling while maintaining three-dimensional cellular architecture, linking oxidative stress mitigation, mitochondrial recovery, and pathway activation.

**Figure 10:**
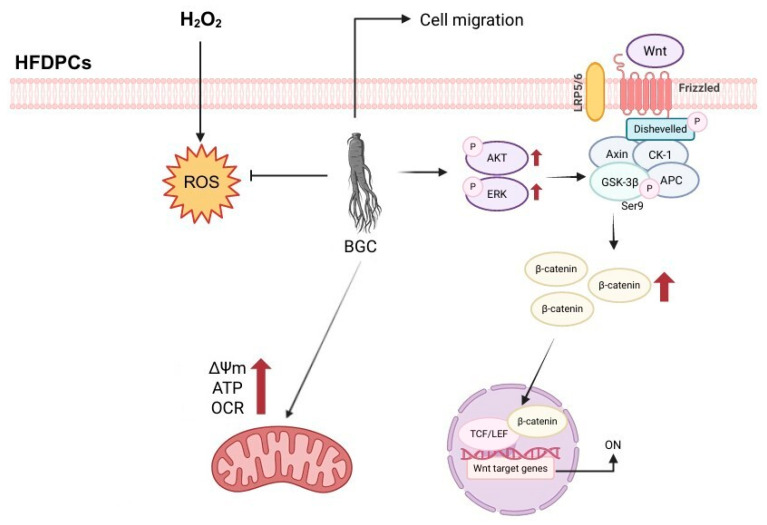
Schematic illustration of the proposed mechanism by which BGC restores hair growth-related signaling in HFDPCs. BGC reduces intracellular ROS levels, restores mitochondrial function, and suppresses oxidative stress-induced signaling dysregulation in HFDPCs. In addition, BGC enhances hair growth-related cellular functions by restoring Wnt/β-catenin signaling and activating the ERK/AKT pathway, thereby preserving dermal papilla cell activity and supporting hair growth under oxidative stress conditions. (Created in BioRender.com. (2026) (https://www.biorender.com/4x1jdps, accessed on 23 June 2026)).

Taken together, our results suggest that BGC protects HFDPCs from oxidative stress through a multifaceted mechanism, integrating antioxidant defense, the restoration of mitochondrial function, and the enhancement of hair growth-associated signaling pathways. The consistency of findings between 2D and 3D models reinforces the physiological relevance of our data, linking oxidative stress mitigation, mitochondrial recovery, and pathway activation into a unified mechanism (Figure 10). The beneficial effects observed in this study may be related to the bioactive ginsenosides present in BGC. Moreover, BGC is a complex mixture, and the specific contributions of its individual bioactive components remain to be elucidated [84,85]. Major ginsenosides are converted into less polar derivatives during the repeated steaming and drying processes used to produce black ginseng, resulting in the formation of compounds such as Rg3, Rg5, and Rk1, which exhibit enhanced biological activities [51].

Previous studies have suggested that several of these ginsenosides promote hair growth by modulating oxidative stress responses and hair growth-related signaling pathways [86,87]. In particular, ginsenoside Rg3 and compound K have been reported to stimulate hair growth and support dermal papilla cell function [88,89]. Our HPLC analysis further confirms that BGC contains significantly higher concentrations of rare ginsenosides, specifically Rg3, Rg5, and Rk1, compared to conventional red ginseng (Figure S6 and Table S1). Notably, the potent protective and restorative effects of BGC observed in H_2_O_2_-damaged HFDPCs represent more than just the sum of individual components. Unlike previous studies focusing on a single ginsenoside, the multi-component profile of BGC, enriched through our patented HPP-integrated process, likely exerts a superior synergistic effect. This collective bioactivity provides a distinct pharmacological advantage in simultaneously modulating multiple hair growth-related pathways, marking a significant advancement over existing single-constituent approaches. Nevertheless, the present study was conducted entirely in vitro, and future studies will focus on identifying the specific active molecules within BGC and conducting human clinical trials to confirm the hair-loss-alleviating effects in a broader population.

In conclusion, our findings demonstrate that BGC safeguards the physiological integrity of HFDPCs against oxidative damage, thereby sustaining their capacity to support hair follicle development. These results provide a scientific basis for the development of BGC as a cosmetic material targeting oxidative stress-related hair disorders.

## 4. Materials and Methods

### 4.1. Preparation of BGC

BGC was prepared from fresh roots of *Panax ginseng* C.A. Meyer (4–6 years old). The fresh ginseng was subjected to repeated steaming and drying cycles to produce black ginseng. Briefly, the ginseng roots were steamed at 98 °C for 3 h, followed by drying at 50 °C until the desired moisture content was reached. This steaming-drying process was repeated three times, which was optimized in conjunction with subsequent high-pressure processing (HPP) to achieve the desired ginsenoside profile. The processed black ginseng was then treated using HPP at 600 MPa for 1 min at room temperature to facilitate the conversion of bioactive compounds. After HPP treatment, the material was extracted using a proprietary extraction method based on Korean Patent No. 10-2295116. The resulting extract was then concentrated under reduced pressure to increase the solid content, yielding BGC. BGC used in this study was supplied by Hanbit Flavor & Fragrance Co., Ltd. (Suwon-si, Republic of Korea).

### 4.2. Cell Culture

HFDPCs (a 46-year-old Caucasian female donor) obtained from PromoCell (Heidelberg, Germany) were cultured in dermal papilla cell growth medium supplemented with the recommended growth factor mix and penicillin-streptomycin (1%) at 37 °C in a 5% CO_2_ incubator. For cell maintenance, the ready-to-use HFDPC Medium and DetachKit (PromoCell, Heidelberg, Germany) were employed according to the manufacturer’s instructions. The detachKit, consisting of HEPES-buffered BSS, Trypsin/EDTA solution, and a trypsin neutralization solution, was used for subculturing. Cells were passaged upon reaching 80–90% confluence, approximately every 3 days, by detachment with the DetachKit and subsequent transfer to 150 mm cell culture plates. To minimize functional alterations associated with extended passaging, all experiments were performed using cells at low passage numbers (passage 3–5), and efforts were made to use comparable passage numbers within each experiment to minimize variability.

### 4.3. Cell Viability Assay

Cell Viability was evaluated using the EZ-Cytox Cell Viability Assay Kit (DoGenBio, Seoul, Republic of Korea). HFDPCs were seeded into 96-well plates at 1 × 10^4^ cells/well and incubated for 24 h. Then, the cells were treated with BGC at concentrations of 25, 50, 100, 200, and 400 μg/mL for an additional 24 h. Subsequently, 10 µL of EZ-Cytox reagent was added to each well, followed by 100 µL of dermal papilla cell growth medium, and the plates were incubated at 37 °C for 30 min. The absorbance was recorded at a wavelength of 450 nm by using a Synergy HTX microplate reader (BioTek, Winooski, VT, USA).

### 4.4. Wound Healing Assay

HFDPCs were seeded into 6-well plates (1 × 10^5^ cells/well) and incubated for 48 h until they reached approximately 90% confluence. A straight scratch was made across the center of the cell monolayer using a sterile 200 µL pipette tip. The cells were gently washed once with DPBS to remove detached cells, and fresh medium was added. The cells were treated with 200 µM H_2_O_2_, 5 µg/mL biotin, or BGC at 25 and 50 μg/mL. Wound closure was assessed at 0 and 24 h after scratch induction. Images of both treated and untreated control groups were acquired by using a phase-contrast microscope (ECLIPSE Ts2, Nikon, Tokyo, Japan).

### 4.5. Alkaline Phosphatase Staining (ALP) Assay

HFDPCs were plated into 24-well plates containing 500 µL of culture medium per well. The cells were treated with 200 µM H_2_O_2_, 5 µg/mL biotin, or BGC at 50 μg/mL. For staining, 1 × PBS-T was prepared using 0.05% Tween 20. A fixing solution (0.4 mL) was added to each well, and the wells were incubated for 10 min at room temperature. After fixation, the solution was removed, and the cells were washed twice with 500 µL of 1 × PBS-T. Following the final wash, the residual buffer was aspirated. 250 µL of ALP staining solution (purple) (Abcam, Cambridge, UK) was added to each well, and incubated for 24 h in a humidified incubator under light-protected conditions. After incubation, the cells were rinsed with DPBS. Purple-stained colonies were counted and compared with unstained colonies under a light microscope (ECLIPSE Ts2, Nikon, Tokyo, Japan).

### 4.6. Measurement of Intracellular ROS

HFDPCs were seeded at a density of 6 × 10^4^ cells/well in confocal dishes and incubated for 24 h. The cells were then treated individually with 5 µg/mL biotin or BGC at 50 μg/mL for 22 h, followed by exposure to 200 µM H_2_O_2_ for 2 h. After treatment, the cells were washed with DPBS and then incubated with 10 µM 2′,7′-dichlorofluorescin diacetate (DCF-DA; DCFDA Cellular ROS Assay Kit, Cat No. ab113851; Abcam, Cambridge, UK) in growth medium for 20 min. Following the removal of the staining solution, the cells were washed with DPBS. Then, 2 mL of DPBS was added to each dish for fluorescence measurement. FITC and DIC images were acquired using an Eclipse Ti2 live-cell fluorescence microscope (Nikon, Tokyo, Japan).

### 4.7. Measurement of Membrane Potential in Mitochondria

Mitochondrial membrane potential was assessed using the JC-1 Mitochondrial Membrane Potential Assay Kit (Abcam, Cambridge, UK). HFDPCs were plated into confocal dishes and cultured for 24 h. Cells were treated with 5 µg/mL biotin or BGC (50 μg/mL) for 22 h, followed by stimulation with 200 µM H_2_O_2_ for an additional 2 h. After treatment, the cells were incubated with 5 µM JC-1 dye for 30 min under light-protected conditions. The cells were subsequently washed with DPBS, and fluorescence images were acquired using a Nikon Eclipse Ti2 live-cell fluorescence microscope (Tokyo, Japan).

### 4.8. Live Cell ATP Assay

Mitochondrial ATP production was evaluated using the ATP Red™ and MitoLite™ Green FM Kits (AAT Bioquest, Pleasanton, CA, USA). HFDPCs were cultured for 24 h in a humidified incubator containing 5% CO_2_ at 37 °C. The cells were then treated with 5 µg/mL biotin or BGC (50 μg/mL) for 23 h, followed by additional exposure to 200 µM H_2_O_2_ for 1 h. After treatment, the cells were stained with ATP Red™ working solution for 30 min. The cells were subsequently washed with DPBS and incubated with MitoLite™ Green FM solution for an additional 30 min. Following a final wash with DPBS, the cells were subjected to fluorescence imaging. Fluorescence signals were acquired using a Nikon live-cell fluorescence imaging microscope (Tokyo, Japan).

### 4.9. Oxygen Consumption Rate Analysis

HFDPCs were seeded into 96-well plates at 1 × 10^4^ cells/well. To induce a reduction in cellular oxygen consumption, the cells were treated with 200 µM H_2_O_2_. Subsequently, cells were treated with BGC (50 μg/mL), and 45 min later, cellular bioenergetic parameters were assessed using a Seahorse XF Analyzer (Agilent Technologies, Santa Clara, CA, USA). During the assay, oligomycin, FCCP, and rotenone/antimycin A were sequentially injected at the designated time points according to the manufacturer’s protocol to modulate mitochondrial respiration. The oxygen consumption rate (OCR) was measured to evaluate cellular energy metabolism.

### 4.10. Western Blot Analysis

HFDPCs were cultured in 100 mm culture dishes and incubated for 24 h. Then, cells were treated with 5 µg/mL biotin or BGC (25 and 50 μg/mL) for 22 h, followed by additional exposure to 200 µM H_2_O_2_ for 2 h. After treatment, the cells were washed with DPBS, and total protein was extracted using RIPA buffer. The lysates were subjected to centrifugation at 12,000× *g* rpm for 10 min. The supernatants were collected, and the total protein concentration was determined using the Pierce™ BCA Protein Assay Kit (Thermo Fisher Scientific, Waltham, MA, USA). Equal amounts of protein were mixed with 4 × LDS sample buffer diluted in RIPA buffer and heated at 70 °C for 10 min. After electrophoresis and transfer, the membranes were blocked with 5% non-fat dry milk in TBS-T containing 0.1% Tween 20. The membranes were then incubated overnight at 4 °C with primary antibodies: phospho-ERK (1:1000; Cat No. 9101S), ERK (1:1000; Cat No. 9102S), phospho-AKT(1:1000; Cat No. 4060S), AKT(1:1000; Cat No. 9272S), all purchased from Cell Signaling Technology (Danvers, MA, USA). Additionally, antibodies against phospho-GSK-3β (Ser9) (1:500; Cat No. sc-373800), GSK-3β (1:500; Cat No. sc-377213), and β-catenin (1:500; sc-59737) were obtained from Santa Cruz Biotechnology (Dallas, TX, USA). Following washing with TBS-T, the membranes were incubated with appropriate secondary antibodies for 2 h at room temperature. Immunoreactive bands were detected using ECL reagent (1:1), and images were captured with an Invitrogen iBright 1500 imaging system (Thermo Fisher Scientific, Waltham, MA, USA). The intensity of the protein bands was quantified using ImageJ software (National Institutes of Health, Bethesda, MD, USA). β-catenin expression was normalized to β-actin, whereas the phosphorylation levels of ERK, GSK-3β, and AKT were normalized to their respective total protein levels.

### 4.11. 3-Dimensional Spheroid Immunofluorescence Analysis

HFDPCs were seeded at a density of 5 × 10^4^ cells/well in 96-well plates with round-bottom and cultured for 2 weeks at 37 °C in a 5% CO_2_ incubator to allow spheroid formation. The spheroids were then treated with 5 µg/mL biotin or BGC (25 and 50 μg/mL) for 22 h, followed by additional exposure to 200 µM H_2_O_2_ for 2 h. After treatment, the spheroids were fixed with 4% paraformaldehyde for 10 min. Then, the spheroids were permeabilized with 0.1% Triton X-100 (Sigma-Aldrich, St. Louis, MO, USA) for 15 min. Subsequently, the spheroids were blocked with 3% BSA in 0.1% PBS-T for 1 h at room temperature and incubated overnight at 4 °C with a primary antibody against β-catenin. The spheroids were washed with 0.1% PBS-T and incubated with an appropriate secondary antibody for 1 h at room temperature. The nuclei were stained with DAPI (1 µM; Sigma-Aldrich, St. Louis, MO, USA) for 15 min. After a final wash with DPBS, fluorescence images were acquired using a Nikon live-cell fluorescence imaging microscope (Tokyo, Japan).

### 4.12. HPLC Analysis for Ginsenosides

To prepare the samples, 1 g of the samples was extracted with 50 mL of 70% methanol for 15 min using a sonicator. The extracts were then filtered through a 0.50 μm syringe filter. The content and the composition of the ginsenoside were analyzed by using HPLC (Agilent 1200 Series, Santa Clara, CA, USA) equipped with a UV-Vis detector. Chromatographic separation was performed on an Eclipse XDB-C18 column (4.6 mm× 250 mm, 5 μm particle size) maintained at a constant temperature of 30 °C. The mobile phase consisted of water (solvent A) and acetonitrile (solvent B). For the gradient elution system, the following program was applied: 0~10 min (20% B), 10~20 min (22% B), and 20~30 min (25% B), 30~38 min (30% B), 38~50 min (38% B), 50~55 min (45% B), 55~70 min (60% B), 70~75 min (60% B), 75~77 min (60% B), 77~80 min (90% B), 80~85 min (20% B), and 85~95 min (20% B). The flow rate was set at 0.9 mL/min, and the injection volume was 10 μl. The detection wavelength was monitored at 203 nm. To identify and quantify the diverse yet structurally similar ginsenosides, high-purity (98%) reference standards were purchased from Chengdu Biopurify Phytochemicals Co., Ltd. (Chengdu, China). Individual ginsenosides in the samples were identified by directly comparing their retention times (t_R_) with those of the corresponding authentic standards analyzed under identical chromatographic conditions. For quantification, five-point linear calibration curves (r^2^ > 0.999) were constructed for each ginsenoside standard over a concentration range of 10–500 μg/mL, and the quantitative values were calculated based on the respective peak areas.

### 4.13. Statistical Analysis

Results are expressed as the mean ± standard deviation (SD), where error bars represent the SD derived from independent trials. Statistical significance among groups was determined by one-way analysis of variance (ANOVA), followed by an appropriate post hoc test. All analyses were conducted using GraphPad Prism software (version 8.01; San Diego, CA, USA).

## 5. Conclusions

In conclusion, BGC protects HFDPCs from oxidative stress-induced cellular dysfunction and signaling alterations. Therefore, BGC may serve as a potential natural candidate for alleviating hair loss.

## Data Availability

The original contributions presented in this study are included in the article/Supplementary Materials. Further inquiries can be directed to the corresponding author.

## Supplementary Materials

The following supporting information can be downloaded at: https://www.mdpi.com/article/10.3390/ijms27135889/s1.

## Abbreviations

HPP	High-Pressure Processing
BGC	Black Ginseng Concentrate
HFDPCs	Human Follicle Dermal Papilla Cells
H_2_O_2_	Hydrogen peroxide
ALP	Alkaline phosphatase
DCFDA	2′,7′-dichlorodihydrofluorescein diacetate
ROS	Reactive oxygen species
FITC	Fluorescein isothiocyanate
JC-1	5,5′,6,6′-tetrachloro-1,1′,3,3′-tetraethylbenzimidazolocarbo-cyanine iodide
ATP	Adenosine Triphosphate
BCA	Bicinchoninic acid
ERK	Extracellular signal-regulated kinase
GSK3β	Glycogen synthase kinase-3 beta
OCR	Oxygen consumption rate
