# Supplementary material for: Black Ginseng Concentrate Restores Hair Loss-Associated Dysfunction in Human Follicle Dermal Papilla Cells
Source: Int J Mol Sci. 2026 Jun 30;27(13):5889. doi: 10.3390/ijms27135889 (PMC13362445; doi:10.3390/ijms27135889)
Supplement: Supplementary file 1 [file ijms-27-05889-s001.zip › ijms-4223224-supplementary.pdf]

Supplementary Figures

Title: Black Ginseng Concentrate Restores Hair Loss-Associated Dysfunction in Human Follicle Dermal Papilla Cells via Redox Regulation and Wnt/β-Catenin Signaling

Authors: Jung Un Shin[1], Yun Hoo Jo[1], Minha Kim[1], Ki Soo Kim[2], Byeong Bae Jeon[2], Uk Sun JUNG[2], Ki Hyun KIM[2], Eui Soon Kim[2], Chulwan Kim[3], Seung Hwan Lee[3], Dong Wook Shin[1*]

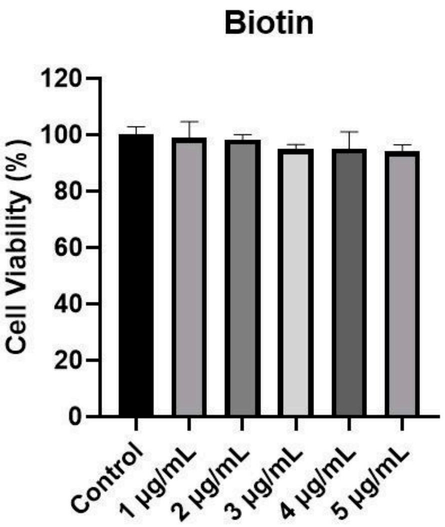

Figure S1. Effect of Biotin on Cell Viability in HFDPCs. HFDPCs were treated with various concentrations of biotin (1-5 μg/mL) for 24 h, and viability was measured using the EZ-cytox assay (absorbance at 450 nm). This concentration range was selected based on a preliminary experiment [1]. In addition, biotin has been reported to modulate hair-related cellular functions in HFDPCs, supporting its biological relevance in this model [2-4]. Since cell viability remained stable across the 1-5 μg/mL range, biotin was determined to be non-cytotoxic to HFDPCs under these conditions. Accordingly, 5 μg/mL was chosen for subsequent studies to evaluate its biological effects while ensuring optimal cell viability. Values represent the mean ± SD from three replicates, normalized to the untreated cells. Statistical analysis indicated no significant differences (ns) compared to the control group.

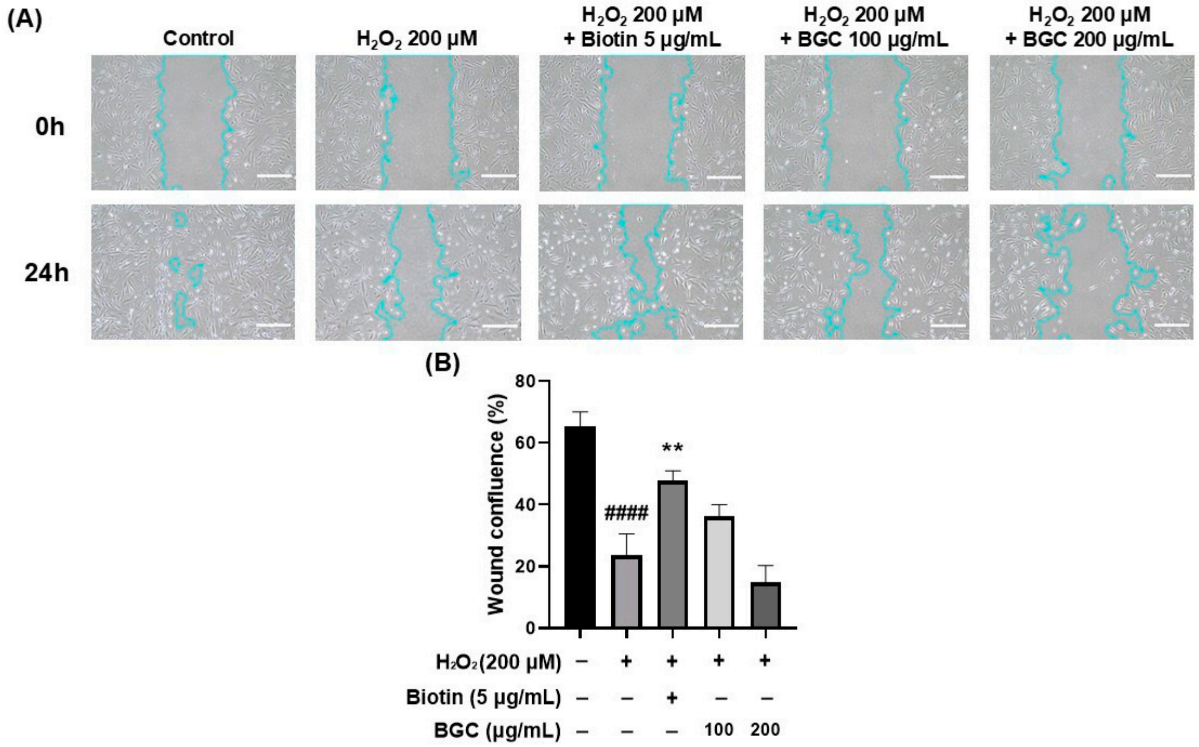

Figure S2. BGC at Concentrations exceeding 100 μg/mL inhibited the migration of H_2O_2-treated HFDPCs. Cells were co-treated with 200 μM H_2O_2 and either 5 μg/mL biotin or BGC (100 and 200 μg/mL) for 24 h. (A) Representative images were obtained using a phase-contrast microscope (scale bar, 50 μm). (B) Quantification of the wound-closure area was performed using ImageJ (version 1.53e). Values represent the mean± SD from three independent biological experiments (n=3). Statistical significance is indicated as $^{**}p < 0.01$ compared with the control group and $^{\#\#\#\#}p < 0.0001$ compared with the control group. No significant difference was observed between the H_2O_2-treated group and the BGC-treated group.

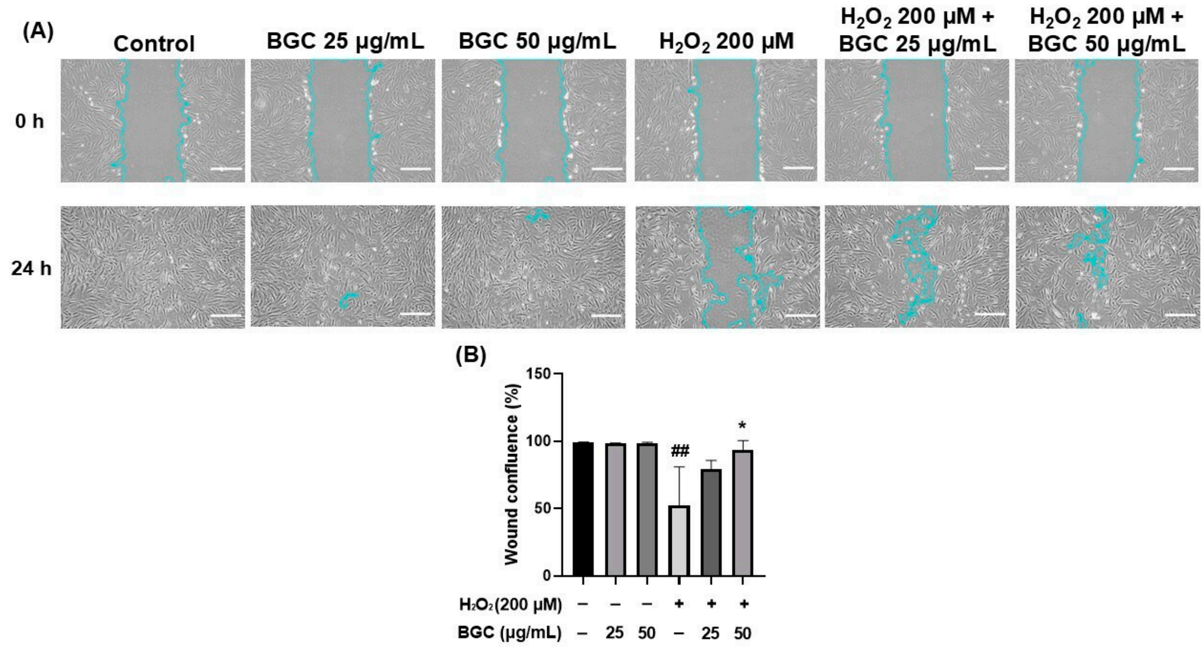

Figure S3. BGC enhances the migratory ability of HFDPCs under oxidative stress. Cells were treated with BGC (25 or 50 μg/mL) alone or in combination with 200 μM H_2O_2 for 24 h. Control and H_2O_2-only groups were included for comparison. (A) Representative images were obtained using a phase-contrast microscope (scale bar, 50 μm). (B) Quantitative analysis of the wound-closure area was conducted using ImageJ software (version 1.53e). Values represent the mean ± SD from three independent biological experiments (n=3). Statistical significance is indicated as $^{*}p < 0.05$ compared with the H_2O_2-treated group and $^{\#\#}p < 0.01$ compared with the control group.

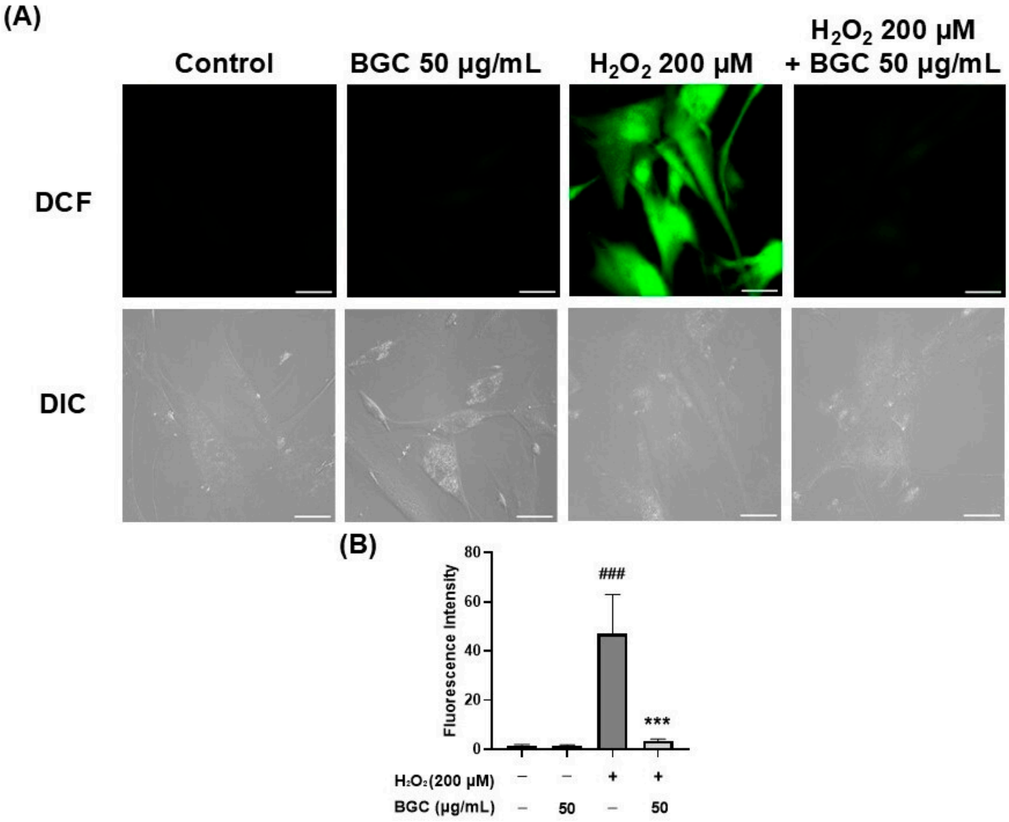

Figure S4. BGC attenuated intracellular ROS levels in H_2O_2-treated HFDPCs under oxidative stress. Cells were treated with BGC (50 μg/mL) for 22 h and then co-treated with 200 μM H_2O_2 for 2 h. (A) Representative DCF-DA fluorescence images showing intracellular ROS accumulation (DCF, green fluorescence, FITC channel) along with DIC images for cell morphological reference (scale bar, 50 μm). (B) Quantitative analysis of relative fluorescence intensity was performed using ImageJ software (version 1.53e). Values represent the mean ± SD from three independent biological experiments (n=3). Statistical significance is indicated as $^{***}p < 0.001$ compared with the H_2O_2-treated group and $^{###}p < 0.001$ compared with the control group.

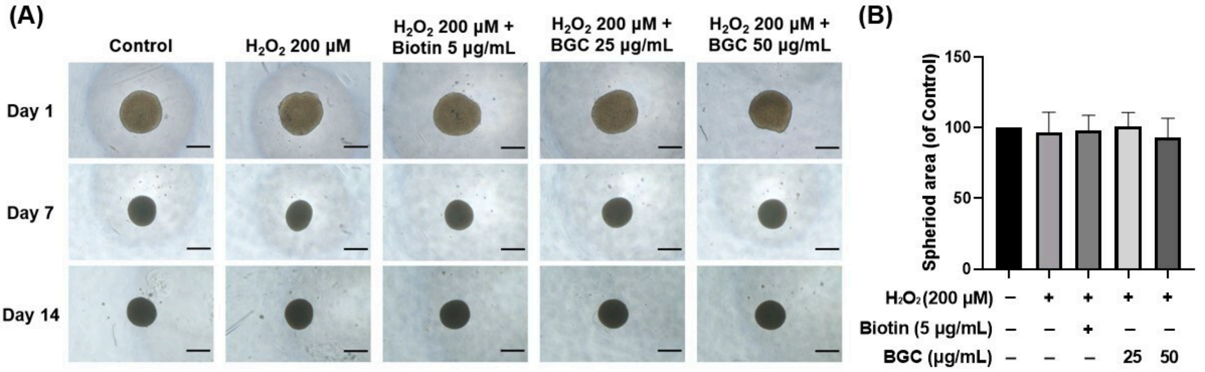

Figure S5. Effect of BGC on the Size of HFDPC 3D Spheroids. HFDPCs were cultured as 3D spheroids to mimic the follicle-like microenvironment. Spheroids were treated with 5 μg/mL biotin or 50 μg/mL BGC in the presence of 200 μM H_2O_2 for 2 weeks. (A) Representative images of 3D spheroids (scale bar, 50 μm). Images are representative of three independent experiments. (B) Quantification of 3D spheroid sizes. Data are expressed as the mean ± SD (n=3). No significant difference was observed between the control group and the H_2O_2-treated group.

a*. Standard

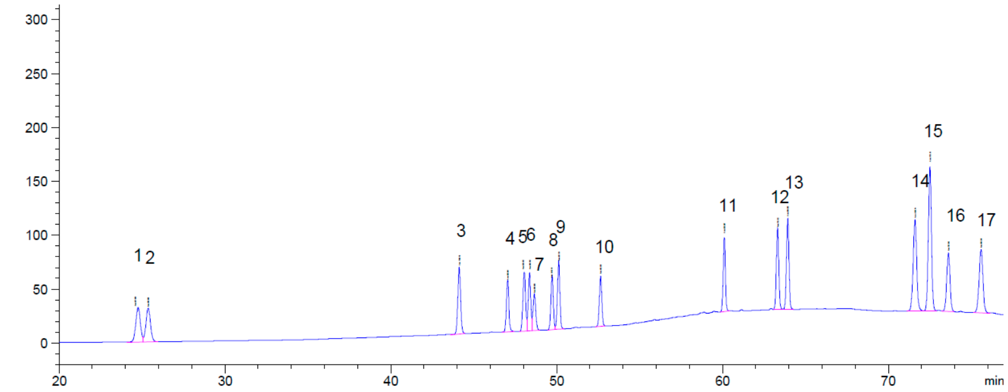

b. Red Ginseng

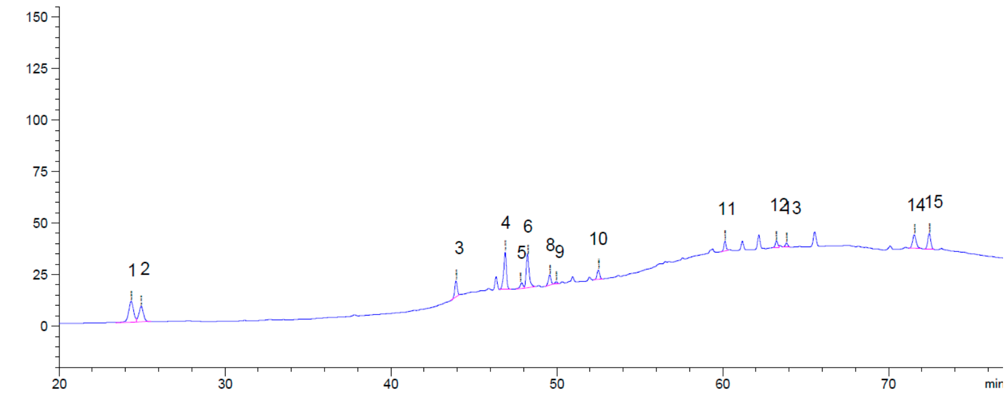

c. BGC

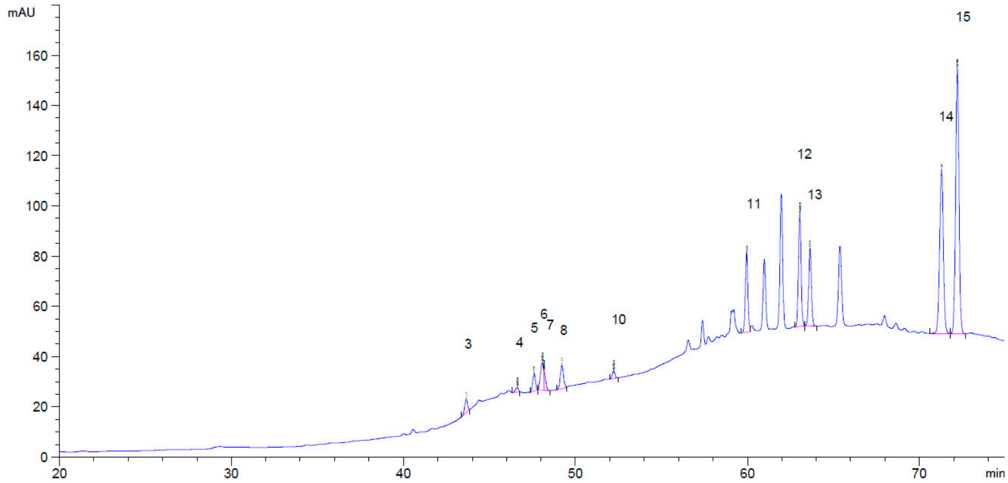

* standard

1: Rg1, 2: Re, 3: Rf, 4: Rb1, 5: Rg2(S), 6: Rc, 7: Rg2(R), 8: Rb2, 9: Rb3,

10: Rd, 11: F2, 12: Rg3(S), 13: Rg3(R), 14: Rk1, 15: Rg5+PPT, 16: CK, 17: Rh2

Figure S6. Comparative HPLC chromatograms of red ginseng and BGC. The profiles illustrate the transition of ginsenoside compositions during the manufacturing process. Peaks were identified by comparing retention times with standard compounds, including Rg3, Rg5, and Rk1. BGC exhibits a significantly higher enrichment of these bioactive minor ginsenosides compared to red ginseng.

Table S1. Comparison of the ginsenosides between red ginseng and BGC.

(units: mg/g)

Ginsenosides	Red Ginseng	BGC
Rg1	2.321±0.009	ND
Re	1.636±0.004	ND
Rf	0.687±0.004	1.249±0.015
Rb1	3.126±0.005	1.636±0.007
Rg2(S)	0.328±0.007	2.136±0.016
Rc	1.860±0.010	3.317±0.001
Rg2(R)	ND	1.221±0.016
Rb2	1.354±0.010	2.386±0.004
Rb3	0.183±0.006	0.083±0.002
Rd	0.372±0.006	1.247±0.004
F2	0.158±0.004	4.673±0.14
Rg3(S)	0.119±0.014	5.192±0.005
Rg3(R)	0.079±0.003	3.684±0.014
Rk1	0.126±0.005	7.166±0.013
Rg5 + PPT	0.105±0.005	11.628±0.010

ND: Not Detected

PPT: Protopanaxatriol
